# Does Supplemental Nutrition Assistance Program Reduce Food Insecurity among Households with Children? Evidence from the Current Population Survey

**DOI:** 10.3390/ijerph18063178

**Published:** 2021-03-19

**Authors:** Jun Zhang, Yanghao Wang, Steven T. Yen

**Affiliations:** 1 School of Agricultural Economics and Rural Development, Renmin University of China, Beijing 100872, China; 2 Global Data Insight and Analytics, Ford Motor Company, Dearborn, MI 48126, USA; wang4348@umn.edu; 3 School of Economics, Jianxi University of Finance and Economics, Nanchang 330013, China

**Keywords:** SNAP participation, food insecurity, ordered probability model, household with children

## Abstract

The Supplemental Nutrition Assistance Program (SNAP) is designed to improve household diet and food security—a pressing problem confronting low-income families in the United States. Previous studies on the issue often ignored the methodological issue of endogenous program participation. We revisit this important issue by estimating a simultaneous equation system with ordinal household food insecurity. Data are drawn from the 2009–2011 Current Population Survey Food Security Supplement (CPS-FSS), restricted to SNAP-eligible households with children. Our results add to the stocks of empirical findings that SNAP participation ameliorates food insecurity among adults only, but increases the probabilities of low and very low food security among children. These contradictory results indicate that our selection approach with a single cross section is only partially successful, and that additional efforts are needed in further analyses of this complicated issue, perhaps with longitudinal data. Socio-demographic variables are found to affect food-secure households and food-insecure households differently, but affect SNAP nonparticipants and participants in the same direction. The state policy tools, such as broad-based categorical eligibility (BBCE) and simplified reporting, can encourage SNAP participation and thus ameliorate food insecurity. Our findings can inform policy deliberations.

## 1. Introduction

The primary goal of the Supplemental Nutrition Assistance Program (SNAP), formerly the Food Stamp Program, is to improve household diet and food security by providing food assistance via benefit payments to households meeting eligibility criteria [1]. In the United States (U.S.), Food security, in which the U.S. Department of Agriculture (USDA) plays a leading role, means access by all people at all times to enough food for an active, healthy life [2]. In 2019, SNAP provided benefits to 35.7 million people in the U.S., with a total federal expenditure of USD 55.6 billion [3]. In addition to SNAP, other food assistance programs such as the National School Lunch Program, Special Supplemental Nutrition Program for Women, Infants, and Children (WIC), and informal food assistance programs are implemented as well. Despite the large government spending, the proportion of households claiming food insecurity has remained high, with 10.5% (13.7 million) of U.S. households being food-insecure at some time during 2019, including 6.4% (8.3 million) with low food security, and 4.1% (5.3 million) with very low food security [2].

Food insecurity has been found to be associated with a wide range of health outcomes, including birth defects, anemia, lower nutrient intakes, cognitive problems, aggregation and anxiety, asthma, behavioral problems, depression, suicide ideation, worse oral health in children, general health, mental health problems and depression, diabetes, hypertension, hyperlipidemia, and lower nutrient intakes among adults [4,5,6,7]. It has remained one of the most pressing problems confronting low-income families today. To fight against food insecurity more effectively, a thorough understanding of its causes and recipients’ responses to food assistance programs is needed for policy deliberations. Some studies have suggested that participation in SNAP increases food insecurity [8,9,10], while insignificant relations between SNAP participation and food security have also been reported [11,12]. A few studies conclude that SNAP participation alleviates food insecurity to some degree [13,14,15,16,17,18,19]. Although most existing studies use data from household surveys which are subject to reporting errors [20,21], a recent study suggests that the degree of reporting error is small and yields relatively little effect on the estimated impact of SNAP participation on food insecurity [21].

One set of analyses on the topic addresses the self-selection of SNAP participation, and finds that participation in SNAP generally ameliorates food insecurity [15,16,18,19,22,23,24,25]. Another set of studies feature uses of the Current Population Survey Food Security Supplement (CPS-FSS) data with longitudinal designs, or augmented by natural or quasi experiments in SNAP effect evaluation [10,26,27,28,29]. To date, studies on the effect of SNAP participation on food insecurity can be characterized as extensive, and inconsistent findings have continued to emerge. These inconsistent findings motivated a panel data analysis by Nord and Golla [17], who concluded that self-selection by more food-needy households into SNAP made it difficult to observe the positive effects of the program in survey data. A thorough investigation into such self-selection is called for.

In addition to studies on SNAP participation and food insecurity, some of the literature focusses on the effects of SNAP benefits on food insecurity, which suggest the benefit level would affect household food insecurity as well [30,31,32]. It has been shown that the 2013 SNAP benefit cut significantly threatened the food security of SNAP households [32], and other research suggests a 35% increase in SNAP expenditure would lead to a reduction in food insecurity by approximately 60% among SNAP participants [33].

In terms of research on the effects of SNAP participation on food insecurity among households with children, the literature is relatively limited, and the results are inconsistent. For example, some studies find SNAP participation to be effective in reducing child food insecurity [19,27,34,35], and that SNAP participation has a greater impact on youth than adults in reducing food insecurity [36]. However, other studies suggest that raising SNAP benefits for households with children does not reduce child food insecurity [37,38].

In this study, we re-take the route of the endogenous selection approach, focusing on households with children and considering categorical (ordinal) household food insecurity. We hope to fill the gap of knowledge by focusing on adults’ and children’s food insecurity and further exploring the impact of SNAP on food insecurity among households with children and with different food insecure categories. Importantly, with the passage of the American Recovery and Reinvestment Act (ARRA) of 2009, SNAP benefits increased temporarily by more than USD 40 billion from 2009 to 2014. Further, SNAP caseloads grew significantly between 2007 and 2011 as the recession and lagging economic recovery led more low-income households to qualify and apply for help [39]. We therefore used data from the older, 2009–2011 Current Population Survey Food Security Supplement (CPS-FSS). These data offered a unique time window to evaluate the roles of SNAP in food insecurity during this period. One previous study used the CPS-FSS data of 2010–2011 to explore the effect of SNAP participation on household food security post ARRA [19], but their samples were small and restricted to husband–wife households with children, which excluded other households with children, such as single-parent households. In this study, we fill this important gap in the knowledge by focusing on all SNAP-eligible households with children from 2009–2011. The larger sample is expected to produce more precise estimates of the role of SNAP participation in food insecurity.

## 2. Materials and Methods

### 2.1. Conceptual Framework

The empirical model is motivated by a utility maximization framework. A SNAP-eligible household maximizes utility, as derived from income (*Y*) and leisure (*L*),

(1) U=U(Y,L)

subject to a time constraint

(2) $$L+W=\bar{T}$$

where *W* is work hours and $\bar{T}$ is time endowment for household members. Household income comes from work and participation in SNAP

(3) Y=Y(W,SNAP)

where *SNAP* is a binary indicator of participation in *SNAP*. There is a disutility function, C=C(S), that affects a household’s choice for *SNAP* participation, where *S* is a set of factors, such as state *SNAP* policies, which affect the participation decision of an eligible household. Then, the *SNAP* participation decision of the household can be expressed as

(4) $$P_{SNAP}=U\left(Y_{SNAP=1},L\right)-U\left(Y_{SNAP=0},L\right)-C\left(S\right)$$

A household will participate in *SNAP* if $P_{SNAP}>0$, but otherwise if $P_{SNAP}\leq 0$. 

Assume food insecurity is a function of household income (Y) and a set of economic and demographic variables (Z) such that $FI_{j}=F\left(Y,Z\right).$ Then, maximizing the utility yields the reduced-form equation for household food insecurity:

(5) $$\begin{array}{l} FI_{j}^{*}=F\left(W,SNAP^{P},Z\right)\;\mathrm{if}\;P_{SNAP}>0\\ =F\left(W,SNAP^{NP},Z\right)\;\mathrm{if}\;P_{SNAP}\leq 0 \end{array}$$

where $FI_{j}^{*}$ is the latent household food insecurity index at category *j*; $SNAP^{P}$ equals 1 and $SNAP^{NP}$ equals 0, and the observed $FI_{j}=j$ if $\xi_{j-1}<FI_{j}^{*}\leq \xi_{j}$ where $\xi_{j-1}$ and $\xi_{j}$ are threshold parameters.

### 2.2. The Econometric Procedure

Motivated by the above theoretical model, we estimate a simultaneous (recursive) equation system. Drawing on existing literature with a single cross-section [16,18], we restrict the effect of food insecurity on SNAP participation to be absent and consider a recursive equation system, with latent food insecurity $\left(y_{1}^{*}\right)$ and SNAP participation $\left(y_{2}^{*}\right)$:

(6) $$y_{1}^{*}=\gamma y_{2}^{*}+x^{\prime }\alpha_{1}+u_{1}$$

(7) $$y_{2}^{*}=x^{\prime }\beta_{1}+w^{\prime }\beta_{2}+u_{2}$$

where x and w are sets of exogenous variables with corresponding parameters $\alpha_{1},{\;\beta }_{1},$ $\beta_{2},$ and γ. The error terms of $\left(u_{1},u_{2}\right)$. are assumed to be distributed as a bivariate standard normal with correlation ρ. The reduced forms consist of (7) and

(8) $$y_{1}^{*}=x^{\prime }(\alpha_{1}+{\gamma \beta }_{1})+w^{\prime }\left({\gamma \beta }_{2}\right)+u_{1}^{*}$$

such that $u_{1}^{*}=u_{1}+\gamma u_{2}$ and $\left(u_{1}^{*},u_{2}\right)$ are distributed as bivariate normal with variances $\left(\sigma^{2},1\right)=\left(1+2\rho \gamma +\gamma^{2},\;1\right)$ and correlation $\tau =\left(\gamma +\rho \right)/(1+2\rho \gamma +\gamma^{2}{)}^{1/2}.$ Based on reduced forms (7) and (8), ordinal food insecurity and binary SNAP participation are governed respectively by

(9) $$y_{1}=k\;\mathrm{if}\;\xi_{k-1}<y_{1}^{*}<\xi_{k},\;k=0,1,\ldots ,K$$

(10) $$y_{2}=1\;\mathrm{if}\;y_{2}^{*}>0\;=0\;\mathrm{if}\;y_{2}^{*}\leq 0$$

where the threshold parameters are $\xi_{0}=-\infty ,{\;\xi }_{1}=0$ and $\xi_{K}=\infty$, as in the conventional ordered probit model. Denote the deterministic portions of (8) and (7) as $h^{\prime }\delta_{1}$ and $h^{\prime }\delta_{2}$ respectively, where $h={\left[x^{\prime },w^{\prime }\right]}^{\prime },$ $\delta_{1}={\left[{\left(\alpha_{1}+{\gamma \beta }_{1}\right)}^{\prime },{\gamma \beta }_{2}^{\prime }\right]}^{\prime },$ $\delta_{2}={\left[\beta_{1}^{\prime },\beta_{2}^{\prime }\right]}^{\prime },$ and the cumulative distribution function of the bivariate standard normal is $\Phi_{2}$. The likelihood contribution is the joint probability

(11) $$\begin{array}{c} \mathrm{Pr}\left(y_{1}=k,y_{2}=j\right)=\Phi_{2}\left[\left(\xi_{k}-h^{\prime }\delta_{1}\right)/\sigma ,{\left(-1\right)}^{j+1}h^{\prime }\delta_{2};{\left(-1\right)}^{j}\tau \right]\\ \;\;\;\;\;\;\;\;\;\;\;\;\;\;\;\;\;\;\;\;\;\;\;\;\;\;\;\;\;\;\;\;\;\;\;\;\;\;\;\;\;\;\;\;\;\;\;\;\;\;\;\;\;\;\;\;\;\;\;\;\;\;\;\;\;\;\;\;\;\;\;\;-\Phi_{2}\left[\left(\xi_{k-1}-h^{\prime }\delta_{1}\right)/\sigma ,{\left(-1\right)}^{j+1}h^{\prime }\delta_{2};{\left(-1\right)}^{j}\tau \right],\;j=0,1 \end{array}$$

The sample likelihood function is the product of the probabilities in Equation (11) over the sample observations. We calculate the average marginal effects of continuous (discrete) exogenous variables by differentiating (differencing) the marginal probability of SNAP participation $\mathrm{Pr}\left(y_{2}=1\right)$ and the conditional probabilities $\mathrm{Pr}(y_{2}=1|y_{1}=k),\;k=0,1$. In addition, the treatment effects of SNAP participation conditional on food insecurity $\left(y_{1}>0\right)$ can be calculated as

(12) $$\begin{array}{ll} TE_{k} & =\mathrm{Pr}\left(y_{1}=k|y_{2}=1,y_{1}>0\right)-\mathrm{Pr}\left(y_{1}=k|y_{2}=0,y_{1}>0\right)\\ & =\frac{\mathrm{Pr}\left(y_{1}=k,y_{2}=1\right)}{\mathrm{Pr}\left(y_{2}=1\right)-\mathrm{Pr}\left(y_{1}=0,y_{2}=1\right)}-\frac{\mathrm{Pr}\left(y_{1}=k,y_{2}=0\right)}{\mathrm{Pr}\left(y_{2}=0\right)-\mathrm{Pr}\left(y_{1}=0,y_{2}=0\right)},k=1,2,3 \end{array}$$

Marginal and treatment effects are calculated for all observations and averaged over the sample. For statistical inference, the standard errors of the average marginal and treatment effects are calculated using an approximation procedure known as the delta method.

## 3. Data and Sample

Data are drawn from the 2009–2011 Current Population Survey Food Security Supplement (CPS-FSS). The USDA has collected information annually on food access and adequacy, food spending, and sources of food assistance for the U.S. population since 1995 [22]. The information is collected in an annual food security survey, conducted by the U.S. Census Bureau as a supplement to the nationally representative Current Population Survey [40]. The CPS-FSS is a key source of national and state data from U.S. households regarding food security and the use of food and nutrition assistance programs. The survey covers the U.S. civilian noninstitutionalized population. The basic CPS sample is selected from multiple frames using multiple stages of selection. The sample design is state-based, with the sample in each state being independent of the others. The food security survey asked one adult respondent in each household questions about the experiences and behaviors of household members that indicate food insecurity. The food security status of the household was assigned based on the number of food-insecure conditions reported. Households with very low food security among children were identified by responses to a subset of questions about the conditions and experiences of children. All households with incomes below 185% of the federal poverty threshold were asked questions about the use of federal and community-based food and nutrition assistance programs.

As noted above, the use of the 2009–2011 CPS-FSS allows the investigation of the post 2009 ARRA era. The sample is restricted to SNAP-eligible households with children (HC), SNAP eligibility being an annual household income lower than 130% of the federal poverty level (FPL). After removing observations with missing values for important variables, the study sample consisted of 5677 SNAP-eligible households with children, of whom 3277 (57.7%) participated in SNAP, defined as households with any member(s) receiving SNAP benefits during the past twelve months. Definitions of variables and sample statistics are presented in Table 1.

### 3.1. Food Insecurity and SNAP Participation Measurements

**Figure 1:**
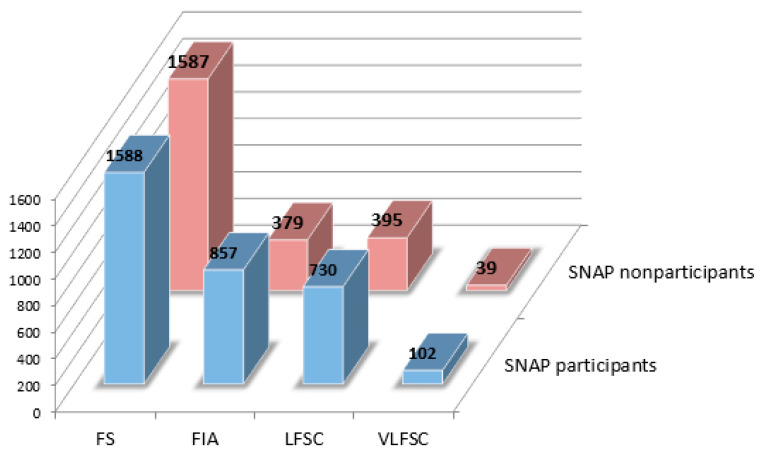
Frequency distribution of SNAP participation and food-insecure categories. FS: food-secure; FIA: food-insecure among adults only; LFSC: low food security among children; VLFSC: very low food security among children.

Household food insecurity is measured based on the responses to 18 items in the Household Food Security Survey Module, 10 of which are specific to the experiences of adults in the household or the household in general, while 8 are specific to the experiences of children under the age of 18 years, during the 12 months prior [41]. Following the USDA’s classifications of food insecurity status for households with children [22,42,43], households’ responses are classified into four categories, according to the number of affirmative answers to the eighteen items and to the eight children-specific items [42]. A household is regarded as (1) food-secure (FS) with less than three affirmative responses to the eighteen food insecure questions; (2) food-insecure among adults only (FIA) with greater than or equal to three affirmative responses to the eighteen food insecure questions, but with less than two affirmative responses to the eight children-specific questions; (3) low food security among children (LFSC) with greater than or equal to three affirmative responses to the eighteen food insecure questions, but with two to four affirmative responses to the eight children-specific questions; (4) very low food security among children (VLFSC) with greater than or equal to three affirmative responses to the eighteen food insecure questions, but with greater than or equal to five affirmative responses to the eight children-specific questions. For food-secure (FS) households, the food insecurity variable is coded as 0, and for food-insecure households, the food insecurity variable ranges from 1 to 3, respectively, for FIA, LFSC, and VLFSC households. Therefore, a higher value of the food insecurity variable corresponds to more severe food insecurity status. SNAP participation is a dummy indicator of anyone in the household receiving SNAP benefits in the past twelve months. Of the SNAP-eligible households, 3277 (58%) are SNAP participants. The two-way frequencies of SNAP participation and food security categories are presented in Figure 1.

### 3.2. Identification Variables

As discussed in previous studies, participation in SNAP is likely to be self-selected and thus endogenous [15,16,17,18,19,22,23,24,25]. The endogeneity of SNAP participation can be addressed with instrumental variable estimation, which requires at least one instrumental variable to be correlated with the endogenous variable but not correlated with the error term in the outcome equation. Due to the distribution assumptions of the error terms in our empirical model, the nonlinear model identification criteria are naturally satisfied without such exclusion restriction(s). However, relying totally on the distributional assumption of the error terms cannot guarantee the identification of model parameters if there are not sufficient sample variations in the exogenous variables. To identify model parameters with sufficient variations, we impose exclusion restrictions with a unique set of variables in the SNAP participation equation.

Three binary-state SNAP policy variables, prevailing in June of the previous year, are natural candidates for tools to assess SNAP participation. Gregory and Coleman-Jensen [44] used similar instruments for their endogenous switching probit model. The one-year lag for SNAP policy variables is appropriate because households’ applications for SNAP are normally submitted several months to a year before the receipt of benefits. The first SNAP policy variable is an indicator of the simplified reporting option, an easier administrative process, for households with earnings, which encourages SNAP participation. The second variable is broad-based categorical eligibility (BBCE) for SNAP. The BBCE policy removes the asset tests for most households and simplifies the application process. Mabli and Ferrerosa [45] found that states that offer a BBCE policy have a 6.2% higher per capita participant count than states without such a policy. The third variable is vehicle test, an indicator of whether the state excludes at least one, but not all, vehicles in the household from the SNAP asset test. Vehicle exemption can lower the bar for SNAP eligibility. Recent studies suggest that vehicle exemption encourages SNAP participation [46].

### 3.3. Socio-Demographic Variables

Drawing on the empirical literature [5,6,19,23], the socio-demographic variables used include respondent’s age, hours of work per week, gender, education level, race, employment status, marriage status, household annual income, number of children, financial needs for food, household size, and locations of residence. In addition, one binary variable was used to indicate whether the HC household is a (primary) husband–wife household, which reveals information about household structure besides marriage status.

## 4. Results and Discussion

### 4.1. Characteristics of Sample

The average age of respondents was 33.41, with a mean of 8.21 work hours per week. About 31% of the respondents were male, 28% did not have a high school diploma, and only 15% had a bachelor’s degree. As regards race, 72% of the households were white, 30% Hispanic, and 20% black. About 55% of the respondents were employed, and one-third were not in the labor force. About 47% were married, and 33% not married. Average household income was USD 16,700 per year, and the mean household size was 4.24, with numbers of children averaging 2.25. About 77% of the households lived in a metropolitan statistical area. The percentages of households living in the south, northeast, west and midwest were 35%, 14%, 27%, and 24%. About 45% were husband–wife households (Table 1).

### 4.2. ML Estimates of Recursive System

The maximum-likelihood estimation of the simultaneous-equation system, and all post-estimation calculations, such as average treatment and average marginal effects, were carried out via programming in MATLAB. The parameter estimates are presented in Appendix A Table A1, and we have summarized the estimates. At the 1% level of significance, all threshold estimates were positive and significant, suggesting the ordered probability model was successful in delineating food-insecure categories. The estimated error correlation was positive and significant at the 1% level, which suggested that unobserved characteristics affect SNAP participation and food insecurity in the same way. Of the 27 explanatory variables in the SNAP participation equation, 16 were significant at the 10% level or lower, including the 2 state SNAP policy identification variables, which rejected the hypothesis of weak instruments. In the food insecurity equation, the endogenous latent SNAP had a significant and negative coefficient at the 5% level, indicating that participation in SNAP can reduce food insecurity. A negative sign of SNAP in the food insecurity equation was reported in previous studies [16,18]. Of the 24 explanatory variables in the food insecurity equation, 11 were significant. To further investigate the effects of SNAP participation on food insecurity, and the effects of the corresponding explanatory variables, the treatment effect of SNAP participation on food insecurity and the marginal effects of explanatory variables are discussed below.

### 4.3. Treatment Effects of SNAP Participation on Food Insecurity

The average treatment effects (ATE) of SNAP participation were calculated, conditional on household’s food insecure status, to further quantify the effects of SNAP participation on food insecurity among food-insecure HC households. The results suggest that, conditional on household’s food insecurity, participation in SNAP decreased the probability of FIA, but increased the probabilities of LFSC and VLFSC (Table 2). For a randomly selected HC household that is food-insecure, participation in SNAP led to a 7.3 percentage points (henceforth, point(s)) lower probability of FIA, but 5.2- and 2.1-points higher probabilities of LFSC and VLFSC, than a nonparticipating household. The signs of treatment effects corroborated those reported by Zhang and Yen [19], who focused on 2010–2011 husband–wife households with children, but our magnitudes are smaller.

### 4.4. Marginal Effects on Probability of SNAP Participation

Table 3 presents the marginal effects on the probability of SNAP participation, based on the SNAP participation Equation (7). Age, work hours, household income, being a husband–wife household, and college education were all deterrents to SNAP participation. The effects were quite notable for these variables—from 4.6 points for work hours to as high as 15.2 points for a HC household. These are primarily variables that improve a household’s economic well-being, which, in turn, alleviates the need for SNAP participation. Older households, for instance, were less likely to participate in SNAP than younger ones, as household economic conditions are likely to improve as a household ages. Husband–wife households were as much as 15.16 points less likely to participate in SNAP than households of other structures. Similar negative factors of SNAP participation have been reported in the literature [18].

The two SNAP policy variables, BBCE and simplified reporting, effectively promoted SNAP participation by as much as 6.37 and 7.28 points, respectively. A need for money for future food consumption also had a notable effect, at 6.69 points. Other positive factors of SNAP participation included household size and number of children. Relative to those who were not in the labor force, both the employed and unemployed had a 4- to 5-points higher probability of participating in SNAP. Regional difference was barely noticeable, with households residing in the midwest being 3.1 points more likely to participate in SNAP than households in the west.

### 4.5. Marginal Effects on Probabilities of FI Categories

The marginal effects, presented in Table 4, suggest that explanatory variables affect the probabilities of food-insecure categories differently between SNAP participants and nonparticipants, but most differences were small in reference to standard errors of the estimates. In this study, we calculate and interpret the marginal effects on the probabilities of FI based on conditional probabilities rather than joint probabilities [19], which are consistent with our conceptual framework and reflect the observed food insecurity status as conditional on SNAP participation.

For instance, conditional on SNAP participation (*viz*., among the participants), a USD 10,000 increase in household annual income would raise the probability of FS by 1.61 points and increase the probability of FIA, LFSC, and VLFSC by 0.57, 0.89, and 0.16 points, respectively. Conditional on nonparticipation, the effect on FS was higher and it was smaller on FIA, with probability increases of 1.96 points and 0.35 points, respectively, and the effect was greater on the probability increases of LFSC (1.22 points) and VLFSC (0.38 point). None of these estimates differed significantly between the SNAP participants and nonparticipants, in that each estimate did not lie outside the 95% confidence interval of the corresponding estimate with a different SNAP participation status.

Besides income, also contributing to food security were male-headed households, college education, residing in the midwest, BBCE, and simplified reporting. College education had especially pronounced effects, with a college-educated household being over 5 points more likely to be food-secure, compared to households headed by an individual with only high school education. This factor affected VLFSC more among SNAP nonparticipants than participants. Conditional on SNAP participation, college education decreased the probabilities of FIA by 1.85 points, LFSC by 2.84 points, and VLFSC by 0.49 points. Conditional on nonparticipation, the estimates were lower for FIA (1.28 points), but higher for LFSC (3.45 points) and VLFSC (0.95 point).

The two state SNAP policy variables, BBCE and simplified reporting, played as definitive roles in combating food insecurity as they did in promoting SNAP participation—the root of the food security improvement. These variables increased the probabilities of FS by over 3 points and decreased the probabilities of food insecurity by between 0.5 and 2.5 points. Simplified reporting decreased the probability of VLFSC by over 2 points. The continued use of these two policy tools in alleviating food insecurity is well justified.

As in SNAP participation, regional differences in food insecurity probabilities are barely noticeable. Compared with those residing in the west, households in the midwest were 3.1 to 3.2 points more likely to be FS, and less likely to be in the food-insecure categories, especially VLFSC (1.73 to 1.97 points). Compared to female-headed households, male-headed households were more likely to be food-secure and less likely to be food-insecure, by 0.33 to 2.06 points. Female-headed households were the vulnerable groups which should be targeted for interventions.

The adverse factors of food security include age, number of children, unemployment, race (white, other race), and financial needs for food purchase. Many of these variables are suggested in the empirical literature [5]. Older households were less likely to participate in SNAP (Table 3) and yet they were more vulnerable to food insecurity. This suggests SNAP participation was not driven entirely by financial conditions, as other factors such as transportation and administrative procedures might deter older households from participating in SNAP, hampering the policy objective of SNAP in alleviating food insecurity among the elderly. Yen et al. [18] also found older households were more vulnerable to food insecurity.

The financial ability to buy food is by far the most notable predictor of household food insecurity. Households in need of money for food acquisition were about 35 points less likely to be food-secure, and were more likely to be in the food-insecure categories (by over 20 points for LFSC). In terms of household structure, neither household size nor being an HW household affected food insecurity. Young children in the household did have an adverse impact on food security, with one additional child decreasing the probability of FS by between 1.59 and 1.74 points, and increasing the probabilities of being food insecure from 0.17 to 1.07 points.

Unemployment can take a toll on household food security. Contrary to common expectations, households of white and other races were less likely to be food-secure and more likely to be in the food insecurity categories than black households. Finally, the negative effects of the year 2009 on the probability of being food-secure, and the positive effects on the probabilities of being food-insecure, echo the statistics of increasing food security over time.

## 5. Conclusions

We investigated the effects of SNAP participation and socio-demographic variables on food insecurity among HC households, using data from a large U.S. national survey. The severity of food insecurity is coded on an ordinal scale, and a recursive ordered probability system is developed to address the endogenous SNAP and ordered food insecurity. Our primary finding is that SNAP participation reduces the probability of being FIA, but increases the probabilities of LFSC and VLFSC. Our results add to the stocks of empirical findings [16,18] that SNAP participation helps households with FIA. Contradictory results of SNAP participation, however, are found on LFSC and VLFSC. Positive effects of SNAP participation on LFSC and VLFSC are possible when taking into account the possibility that households with severe food insecurity are more likely to participate in SNAP. They may also indicate that our selective approach to the evaluation of SNAP’s effects on food insecurity with a single cross-section is only partially successful, and that additional efforts are needed in further analyses of this complicated issue. Successes with the use of longitudinal data, augmented with natural or quasi experiments, as cited above, are encouraging. Whereas the longitudinal data do not make the selection issue go away, they do offer a better data environment for identifying the models with endogenous SNAP participation.

This study is among the first of its kind to focus on HC households in evaluating the implications of SNAP participation in ordinal food insecure categories. Similar existing studies focused on husband–wife households with children, but excluded other types of households with children [19]. Findings can inform the administration, law makers, and the public concerned about household food security issues. By the calculating marginal effects of explanatory variables between SNAP nonparticipants and participants, we found that socio-demographic variables affect food-secure households and food-insecure households differently, but affect SNAP nonparticipants and participants in the same way. For both SNAP-participating and -nonparticipating households, the probabilities of FIA, LFSC, and VLFSC are all higher among respondents who are aged, with lower incomes, more children, less education, who are unemployed, of other races, and have financial needs for food. Our findings also suggest that the state policy tools of BBCE and simplified reporting can encourage SNAP participation and thus ameliorate the probabilities of FIA, LFSC, and VLFSC.

While this paper represents one of the first attempts to investigate the role of SNAP participation in the ordinal food insecurity of children and adults, a few caveats pertain. First, while the 2009–2011 CPS-FSS offered a unique time window to evaluate the roles of SNAP in food insecurity after the passage of the 2009 ARRA, the data are indeed old, and the findings may not reflect the current status of household food insecurity. Further studies might consider the use of more recent data. Second, the current study is based on a single cross-sectional data set, focusing on the role of SNAP; future studies might consider comparing results from panel data investigations and the analysis of other food assistance programs, such as WIC and informal food assistance programs. Finally, SNAP and socio-demographic factors are likely to be important for diet quality and nutrition, and interesting insights may emerge with a similar study on these alternative outcomes.

## Figures and Tables

**Table 1:** Definitions and sample statistics of variables.

Variable	Definitions	Mean	SD
Endogenous variables			
FI	Household food insecurity category: 0 = food secure (FS), 1 = food	0.69	0.87
	insecure among adults only (FIA); 2 = low food security among children (LFSC); 3 = very low food security among children (VLFSC)		
SNAP	Any member(s) in household received SNAP benefits past 12 months	0.58	0.49
Continuous explanatory variables			
Age	Age of respondent	33.41	8.06
Work hours	Respondent’s work hours per week	18.21	19.58
Income	Household annual income in $10,000	1.67	0.97
Household size	Number of persons living in household	4.24	1.56
Children	Number of children < 18 years of age	2.25	1.16
Binary explanatory variables (yes = 1, no = 0)			
State policy variables			
BBCE	State uses broad-based categorical eligibility (BBCE) categorical eligibility for SNAP	0.55	
Simplified reporting	State uses simplified reporting for households with earnings	0.88	
Vehicle test	State excludes at least one, but not all, vehicles in household from SNAP asset test	0.12	
Household characteristics			
Year 2009	Data collected in 2009	0.28	
Year 2010	Data collected in 2010	0.32	
Year 2011	Data collected in 2011 (reference)	0.40	
HW household	Primary husband-wife household	0.45	
MSA	Resides in metropolitan statistical area	0.77	
South	(Reference person) resides in south	0.35	
Northeast	Resides in northwest	0.14	
West	Resides in west (reference)	0.27	
Midwest	Resides in midwest	0.24	
More money	Need to spend more money to buy enough food to meet needs	0.34	
Respondent/reference person characteristics			
Male	Gender is male	0.31	
Married	Married	0.47	
Not married	Not married (reference)	0.33	
Separated	Separated or divorced	0.20	
<High school	<High school education	0.28	
High school	High school graduate (reference)	0.36	
Some college	Attended college but no degree	0.21	
College	Has college education or higher	0.15	
Employed	Employed	0.55	
Unemployed	Unemployed	0.16	
Not in labor force	Not in labor force (reference)	0.30	
Hispanic	Of Hispanic origin	0.30	
White	White	0.72	
Black	Black (reference)	0.20	
Other race	Other race	0.08	
Sample size		5677	

**Table 2:** Average treatment effects (ATE) of SNAP on probabilities of food insecurity conditional on FI > 0.

Food Insecure Category	ATE
Food-insecure among adults (FIA)	−0.073 (0.008)
Low food security among children (LFSC)	0.052 (0.006)
Very low food security among children (VLFSC)	0.021 (0.003)

Note: Asymptotic standard errors are in parentheses. All effects are significant at the 1% level.

**Table 3:** Average marginal effects of explanatory variables on the probability of SNAP participation.

Variable	Probability
Continuous explanatory variables	
Age/10	−6.76 (0.82) ***
Work hours/10	−4.57 (0.57) ***
Income	−11.29 (0.67) ***
Household size	3.51 (0.63) ***
Children	2.30 (0.73) ***
Binary explanatory variables	
Year 2009	−5.22 (1.55) ***
Year 2010	−1.18 (1.45)
Male	0.92 (1.49)
HW household	−15.16 (4.31) ***
<High school	0.17 (1.59)
Some college	−0.74 (1.65)
College	−7.05 (1.85) ***
Employed	5.22 (2.25) **
Unemployed	4.24 (1.96) **
Hispanic	−9.32 (1.56) ***
White	−2.23 (1.77)
Other race	−0.28 (2.63)
MSA	−3.30 (1.45) **
South	−0.04 (1.76)
Northeast	2.97 (2.15)
Midwest	3.11 (1.85) *
Married	0.33 (4.09)
Separated	0.05 (1.84)
More money	6.69 (1.26) ***
BBCE	6.37 (1.34) ***
Simplified reporting	7.28 (2.08) ***
Vehicle test	2.14 (1.78)

Note: All effects on probabilities are multiplied by 100. Asymptotic standard errors are in parentheses. Asterisks indicate level of significance: *** = 1%, ** = 5%, * = 10%.

**Table 4:** Marginal effects of explanatory variables on the probabilities of food-insecure categories, conditional on SNAP participation status.

	Conditional on SNAP Participation				Conditional on SNAP Nonparticipation			
Variable	FS	FIA	LFSC	VLFSC	FS	FIA	LFSC	VLFSC
Continuous explanatory variables								
Age/10	−2.77 (0.76) ***	0.91 (0.26) ***	1.56 (0.43) ***	0.30 (0.08) ***	−2.78 (0.80) ***	0.60 (0.17) ***	1.70 (0.50) ***	0.48 (0.15) ***
Income	1.61 (0.67) **	−0.57 (0.22) **	−0.89 (0.38) **	−0.16 (0.07) **	1.96 (0.70) ***	−0.35 (0.15) **	−1.22 (0.43) ***	−0.38 (0.13) ***
Children	−1.59 (0.69) **	0.54 (0.23) **	0.89 (0.39) **	0.17 (0.07) **	−1.74 (0.73) **	0.35 (0.15) **	1.07 (0.45) **	0.32 (0.13) **
Binary explanatory variables								
Year 2009	−3.20 (1.38) **	1.03 (0.45) **	1.81 (0.79) **	0.35 (0.16) **	−3.27 (1.45) **	0.67 (0.28) **	2.01 (0.90) **	0.59 (0.28) **
Male	3.18 (1.41) **	−1.08 (0.49) **	−1.77 (0.78) **	−0.33 (0.14) **	3.36 (1.50) **	−0.72 (0.33) **	−2.06 (0.92) **	−0.58 (0.26) **
College	5.18 (1.66) ***	−1.85 (0.62) ***	−2.84 (0.90) ***	−0.49 (0.15) ***	5.67 (1.81) ***	−1.28 (0.46) ***	−3.45 (1.09) ***	−0.95 (0.28) ***
Unemployed	−5.27 (1.71) ***	1.67 (0.51) ***	3.00 (0.99) ***	0.60 (0.22) ***	−5.61 (1.75) ***	1.00 (0.28) ***	3.50 (1.11) ***	1.11 (0.38) ***
White	−3.03 (1.52) **	1.02 (0.53) *	1.69 (0.84) **	0.31 (0.16) **	−3.17 (1.63) *	0.68 (0.36) *	1.93 (0.99) *	0.55 (0.29) *
Other race	−7.22 (2.40) ***	2.17 (0.65) ***	4.16 (1.42) ***	0.88 (0.35) **	−7.47 (2.43) ***	1.24 (0.32) ***	4.68 (1.54) ***	1.55 (0.59) ***
MSA	−2.96 (1.35) **	1.01 (0.48) **	1.65 (0.75) **	0.30 (0.13) **	−3.07 (1.45) **	0.67 (0.33) **	1.87 (0.88) **	0.52 (0.24) **
Midwest	3.10 (1.67) *	−1.06 (0.59) *	−1.73 (0.92) *	−0.32 (0.16) *	3.22 (1.79) *	−0.71 (0.40) *	−1.97 (1.09) *	−0.55 (0.30) *
More money	−34.72 (1.22) ***	10.48 (0.53) ***	20.76 (0.91) ***	3.48 (0.37) ***	−35.28 (1.17) ***	4.85 (0.45) ***	23.80 (0.94) ***	6.63 (0.55) ***
BBCE	3.12 (1.05) ***	−1.02 (0.35) ***	−1.76 (0.59) ***	−0.34 (0.12) ***	3.17 (1.11) ***	−0.67 (0.22) ***	−1.94 (0.68) ***	−0.56 (0.21) ***
Simplified reporting	3.55 (1.38) **	−1.11 (0.42) ***	−2.02 (0.79) **	−0.41 (0.17) **	3.61 (1.43) **	−0.71 (0.25) ***	−2.23 (0.89) **	−0.67 (0.29) **

Note: Marginal effects with respect to statistically insignificant variables are omitted. All effects on probabilities are multiplied by 100. Asymptotic standard errors in parentheses. Asterisks indicate level of significance: *** = 1%, ** = 5%, * = 10%.

## Data Availability

Data of this study are publicly available.

## Appendix A

**Table A1:** Maximum-likelihood estimates of recursive system for SNAP participation and household food insecurity.

Variable	SNAP Participation	Food Insecure
Latent SNAP		−0.307 (0.125) **
BBCE	0.194 (0.041) ***	
Simplified reporting	0.220 (0.062) ***	
Vehicle test	0.066 (0.055)	
Year 2009	−0.159 (0.047) ***	0.016 (0.052)
Year 2010	−0.036 (0.045)	−0.031 (0.039)
Age/10	−0.207 (0.025) ***	−0.015 (0.037)
Male	0.028 (0.046)	−0.072 (0.043) *
HW household	−0.447 (0.126) ***	−0.212 (0.121) *
<High school	0.005 (0.049)	0.050 (0.042)
Some college	−0.023 (0.051)	0.028 (0.045)
College	−0.213 (0.056) ***	−0.228 (0.053) ***
Employed	0.163 (0.072) **	0.116 (0.064) *
Unemployed	0.131 (0.061) **	0.192 (0.050) ***
Work hours/10	−0.140 (0.018) ***	−0.070 (0.022) ***
Income	−0.346 (0.022) ***	−0.189 (0.042) ***
HH size	0.108 (0.019) ***	0.042 (0.021) **
Children	0.071 (0.022) ***	0.072 (0.021) ***
Hispanic	−0.281 (0.047) ***	−0.079 (0.056)
White	−0.068 (0.054)	0.052 (0.048)
Other race	−0.009 (0.081)	0.182 (0.069) ***
MSA	−0.101 (0.045) **	0.035 (0.044)
South	−0.001 (0.054)	0.021 (0.045)
Northeast	0.091 (0.067)	0.044 (0.062)
Midwest	0.096 (0.057) *	−0.042 (0.055)
Married	0.010 (0.126)	0.052 (0.107)
Separated	0.002 (0.056)	0.035 (0.049)
More money	0.206 (0.039) ***	0.912 (0.048) ***
Constant	1.112 (0.129) ***	−0.280 (0.245)
Thresholds		
ξ_1_		0.623 (0.047) ***
ξ_2_		1.813 (0.133) ***
Error correlation (ρ)		0.479 (0.114) ***
Log likelihood	−8776.7229	

Note: Asymptotic standard errors in parentheses. Asterisks indicate level of significance: *** = 1%, ** = 5%, * = 10%.
